# Efficient string similarity join in multi-core and distributed systems

**DOI:** 10.1371/journal.pone.0172526

**Published:** 2017-03-09

**Authors:** Cairong Yan, Xue Zhao, Qinglong Zhang, Yongfeng Huang

**Affiliations:** School of Computer Science and Technology, Donghua University, Shanghai, China; Nanjing Normal University, CHINA

## Abstract

In big data area a significant challenge about string similarity join is to find all similar pairs more efficiently. In this paper, we propose a parallel processing framework for efficient string similarity join. First, the input is split into some disjoint small subsets according to the joint frequency distribution and the interval distribution of strings. Then the filter-verification strategy is adopted in the computation of string similarity for each subset so that the number of candidate pairs is reduced before an effective pruning strategy is used to improve the performance. Finally, the operation of string join is executed in parallel. Para-Join algorithm based on the multi-threading technique is proposed to implement the framework in a multi-core system while Pada-Join algorithm based on Spark platform is proposed to implement the framework in a cluster system. We prove that Para-Join and Pada-Join cannot only avoid reduplicate computation but also ensure the completeness of the result. Experimental results show that Para-Join can achieve high efficiency and significantly outperform than state-of-the-art approaches, meanwhile, Pada-Join can work on large datasets.

## 1 Introduction

String similarity join that finds similar string pairs in a given string set or between two given string sets is a fundamental operation in many fields, such as pattern matching, computational linguistics, bioinformatics, and database integration [1].It is widely used for detection of duplicate web pages in web crawling [2], collaborative filtering [3], and entity resolution [4]. For example, given two string sets R = {Mi Li, Qi Wan, …} and S = {M. Li, Qin Wan, …}, we can find all similar pairs <*r*,*s*> ∈ *R* × *S* such as <Mi Li, M Li> according to a certain similarity function.

For string similarity join, fundamental techniques include partitioning techniques (e.g. Pass-Join [5] and PartEnum [1]), prefix-filtering methods (e.g. TrieJoin [6] and PEARL [7]), and other methods (e.g. MTree [8], SSI [9], LSH [10], and FASTSS [11]). Research in this field has been carried out in various scientific disciplines and related methods often are tuned for specific ranges of allowed error thresholds or query lengths, specific hardware properties, specific alphabet sizes, or specific distributions of errors.

The big data era is the inevitable consequence of our ability to generate, collect, and store digital data at an unprecedented scale. When there are a large number of sources and a large volume of data, the traditional string join methods become inefficient and ineffective to practice. To address the volume dimension, new techniques have been proposed to enable parallel string join using MapReduce. These include techniques for adaptive blocking [10] and techniques that balance load among different nodes. However, MapReduce is not adapted for the application of data join.

In this paper, we propose a parallel string join framework to address the efficiency problem by utilizing the multi-threading technique and distributed computing technique separately. Availability of high-performance CPU and the large memory make the framework very practical. The contributions of this paper are as follows:

1. We propose a parallel processing framework for string similarity join and a related pruning strategy to obtain the high efficiency. The partition-based method and the parallel processing techniques are used to improve the computational performance.
2. We propose a parallel string join algorithm Para-Join to implement the framework in the multi-core system. The multi-threading technique is used to improve the processing performance. We demonstrate that Para-Join method can not only avoid reduplicate computation but also ensure the completeness of the result.
3. We propose a parallel string join algorithm Pada-Join to implement the framework in the cluster environment based on Spark platform to obtain the high efficiency.
4. We have implemented and tested Para-Join and Pada-Join algorithms on real data sets. The experimental results show that our algorithm achieves high performance and outperforms the existing methods.

The rest of this paper is organized as follows: Section 2 gives the notations and discussion of some existing techniques. In section 3, we introduce our proposed parallel string similarity join framework and related strategies. Then, section 4 and 5 give the details of Para-Join algorithm and Pada-Join algorithm separately. We present the experimental results in section 6. Related works are introduced in section 7 and we conclude the paper in section 8.

## 2. Background

### 2.1 Formal problem statement

#### Definition 1: String and string set

A string *s* is a finite sequence of symbols over an alphabet *Σ*. The length of *s* is denoted by |*s*| and the substring starting at position *i* with length *n* is denoted by *s*(*i*,*n*). All positions in a sequence are zero-based, i.e., the first character of *s* is *s*(0). A string set *S* is a collection of strings. The size of *S* is denoted by |*S*|.

#### Definition 2: String similarity

Given strings *s* and *r*, *s* is similar to *r*, denoted *s* ∼sim *r*, if and only if *Sim*(*s*,*r*)≥*δ*. *Sim(s*,*r)* is a certain similarity function and *δ* is a threshold. If the edit distance is used as the similarity function, *s* is *k*-approximately similar to *r*, denoted *Ed(s*,*r) = k*, if and only if *s* can be transformed into *r* by at most *k* edit operations. The edit operations include replacing one symbol in *s*, deleting one symbol from *s*, and inserting one symbol into *s*.

$$Sim(s,r)=1-\frac{Ed(s,r)}{Max(|s|,|r|)}\geq \delta$$ (1)

Ed(s,r)≤(1−δ)×Max(|s|,|r|) (2)

τ=(1−δ)×Max(|s|,|r|) (3)

#### Definition 3: String similarity (self) join

Given two string sets *S* and *R*, a similarity function *Sim*() (or an edit distance function *Ed*(·)) and a similarity threshold *δ* (or a distance threshold *τ*), a similarity join finds all string pairs *<s*,*r>* (*s*∈*S*, *r*∈*R*) such that *Sim*(*s*,*r*) ≥ *δ* (or *Ed*(*s*,*r*) ≤ *τ*). When *R* is equal to *S*, it refers to string similarity self-join of *S*. In this paper we adopt the edit distance to measure the similarity, so we use *τ* as the similarity threshold directly.

### 2.2 Filtering and verification framework

#### Length filtering

Length filtering is proposed in reference [9]. The basic idea is that if two strings are similar, the difference of their length cannot be large. For example, if a string s is similar to r, the length of s should be in the range between *|r|-τ* and *|r|+τ* (*τ* is a given similarity threshold). By using the length filtering method, a string set *s* can be partitioned into subsets and in each subset, the strings share the same length. Then it is not necessary to match the strings in different subsets. Length filtering method is used to reduce the amount of candidate pairs for string similarity matching.

#### Position filtering

Considering two strings *s* and *r*, *s* is split into *τ+1* disjoint segments. If *r* is similar to *s*, there must exist a substring of *r* which can match one segment of *s* based on the pigeonhole principle. For any segment *s(i*,*n)*, let *W*_*n*_ denote the set which includes all the segments of *r* with length *n*. We need to check whether *s(i*,*n)* appears in *W*_*n*_. Length filtering method doesn’t consider the position of the segments so that it cannot deal with this matter. Position filtering method provides an effective substring selection strategy to generate *W*_*n*_ [2, 10]. Because the element number in *W*_*n*_ is smaller than that of all the substrings, a lot of unnecessary calculation will be avoided. For example, if the similarity threshold is *τ* and string *r* has a substring *r(i*,*n)* (*1≤i≤4*) which matches *s(j*,*n)*, position filtering method will give the possible start positions of *r(i*,*n)* in string *r*. The strings *r* and *s* will be split into three parts, the same part *r(i*,*n)* and *s(j*,*n)*, the left part *r(0*,*i)* and *s(0*,*j)*, the right part *r(i+n*,*|r|-i-n)* and *s(j+n*,*|s|-j-n)*. If *s* is similar to *r*, we can transform *s* to *r* with *Ed*(*r*,*s*) ≤ *τ*. A straightforward method is that we first transform *r(0*,*i)* to *s(0*,*j)*, then we transform r*(i*,*n)* to *s(j*,*n)*, finally we transform *r(i+n*,*|r|-i-n)* to *s(j+n*,*|r|-j-n)*. The total transformation distance is *Ed*(*r*(0,*i*), *s*(0,*i*)) + *Ed*(*r*(*i*,*n*), *s*(*i*,*n*)) + *Ed*(*r*(*i* + *n*,|*r*| −*i* − *n*), *s*(*i* + *n*,|*s*| −*i* − *n*)) ≤ *τ*. Based on the above conclusions, we can get the range of the start position of r(i,n) [*P*_min_,*P*_*max*_]. According to reference [2], we can get *P*_min_ = max (1, *p*_*i*_ − ⌊(*τ* − Δ)/2⌋) and *P*_max_ = min (|*s*| − |*s*(*i*,*n*)|, *p*_*i*_ − ⌊(*τ* − Δ)/2⌋), where *p*_*i*_ is the start positions of *s(i*,*n)* in string *s*, and Δ = ||*s*| − |*r*||. So we do not need to visit all the substrings of *r*. Reference [2] improved position filtering by using a new substring selection technology called multi-match-aware substring selection which obtained the more accurate values for *P*_*min*_ and *P*_*max*_.

#### Extension-based verification

Given a candidate pair *<s*,*r>*, a simple method to verify whether they are similar is to calculate the edit distance between them. If the edit distance is not larger than *τ*, we can say that s is similar to *r*. A dynamic-programming algorithm can be used to get the edit distance. The time complexity is O(|*s*| × |*r*|). Actually, we only need to check whether the edit distance is less than *τ* instead of getting the absolute value of edit distance. Length-aware verification was proposed to verify the candidate pair based on length pruning [2,10,12]. In order to further reduce the computation, Wang et al. extended this method through putting forward the extension-based verification to calculate the edit distance [2]. This method can reduce the time complexity from O(|*s*| × |*r*|) to O((*τ* + 1) × min(|*s*|,|*r*|)).

In this paper, we apply the filter-verification framework to implement the string similarity join. The length filter method and the position filter method are used for the first phase, and the extension-based verification method is used for the second phase.

## 3. Parallel string join framework

### 3.1 Parallel processing framework

**Fig 1:**
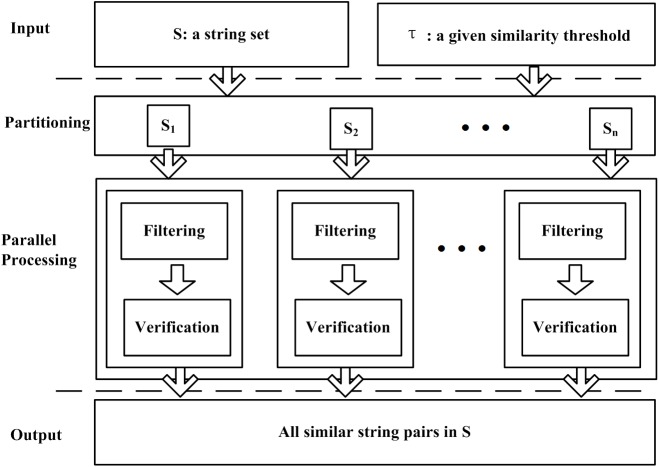
The framework for parallel string similarity join.

Applications continue to become more data-intensive. We assume applications may be pulled apart across the threads in the multi-core system or the nodes in the distributed systems. Although this may complicate data placement and transport, it improves the processing efficiency. Fig 1 shows the proposed parallel string join framework which can benefit from the data parallelism, task parallelism, and resilience. The input includes a string set *S* and a similarity threshold τ. In the second phase, *S* is divided into several partitions. For each partition, a thread or task is created to process it separately. The filter-verification technique is used for string join in each thread or task. Both string partitioning and string matching can be done in parallel. The output is the combination of all the similarity pairs in *S* and is stored in the disk.

In this framework, solutions of three issues are taken into consideration:

- How to split the dataset into subsets. Since the size of the dataset is not determined and the capability of the system is unknown in advance, it is hard to determine the number of the partitions.
- How to calculate the similarity of two strings efficiently. The similarity computation is the core of the framework so we need a more efficient algorithm to deal with it.
- How to implement parallel string join algorithm to obtain high efficiency without affecting the accuracy of string matching. The parallel algorithm must guarantee that the accuracy will not be affected. It will be better to improve the accuracy of the parallel algorithms since the multi-threading and the multi-tasking techniques can reduce the time and space complexity.

### 3.2 String similarity computation

Resume a token set *Σ*, a string set *S*, a string *s*, a string r, *r*∈*S*, and *s*∈*S*. The function *f*^*c*^(⋅) represents the joint frequency vector and function *v*(⋅) represents the interval vector. So *f*^*c*^(*s*) is the joint frequency vector of *s* and *v*(*s*) is the interval vector of *s*. ${\left\|f^{c}(s)-f^{c}(r)\right\|}_{L_{1}}$ is the distance of joint-frequency vector between *f*^*c*^(*s*) and *f*^*c*^(*r*) about length *L*_*1*_. The function *dis*(*v*(*r*),*v*(*s*)) is the distance between *v(s)* and *v(r)*. If the equation *v*(*s*) = *v*(*r*) exists, we can say that *s* and *r* are similar.

According to the above rule, we can split *S* into some disjoint subsets *S*_1_,*S*_2_,⋯,*S*_*n*_, *S*_1_ ⊂ *S*, *S*_2_ ⊂ *S*, *S*_*n*_ ⊂ *S*. Then we can get the following conclusion.

1. *S*_1_∪*S*_2_∪⋯∪*S*_*n*_ = *S*.
2. For each subset *S*_*i*_ and *S*_*j*_, *S*_*i*_ ∩ *S*_*j*_ = Φ, *S*_*i*_ ⊂ *S* and *S*_*j*_ ⊂ *S*.
3. For each subset *S*_*i*_ (*S*_*i*_ ⊂ *S*), if there exist two string *s*_*1*_ and *s*_*2*_ (*s*_1_,*s*_2_ ∈ *S*_*i*_), we can get *v*(*s*_1_) = *v*(*s*_2_).

Table 1 shows an instance of a string set *S*. The size of *S* is 12. Alphabet *Σ* is composed of 26 lowercase letters, i.e., *Σ = {a*, *b*, *…*, *z}*. We divide *Σ* into three subsets, *Σ*_*1*_ *= {g*,*e*,*b*,*n*,*j*,*h*,*w*,*t*,*x}*, *Σ*_*2*_ *= {f*,*a*,*o*,*m*,*k*,*i*,*u*,*q*,*z}*, and *Σ*_*3*_ *= {d*,*c*,*l*,*v*,*s*,*r*,*p*,*y}*. We then further divide *Σ*_*1*_ into four intervals [1,2], [3,3], [4,4], [5,6], divide *Σ*_*2*_ into three intervals [2,2],[3,3],[4,7], and divide *Σ*_*3*_ into four intervals [1,1], [2,2], [3,3], [4,4], [5,6]. First, for each string in *S* we get the joint-frequency vector and the interval-vector by functions *f*^*c*^(⋅) and *v*(⋅).

**Table 1:** An instance of the string set S.

Index	String	Index	String
*s*_*1*_	myfeltypre	*s*_*7*_	rewhoward
*s*_*2*_	cusendusers	*s*_*8*_	ewpalaythe
*s*_*3*_	jeffreyp	*s*_*9*_	toniawbluher
*s*_*4*_	lfredkobs	*s*_*10*_	oldmillerthe
*s*_*5*_	nguisanint	*s*_*11*_	ensgroupwar
*s*_*6*_	dreacutethay	*s*_*12*_	araliskovargu

*f*^*c*^(*s*_1_) = (3,2,5), *f*^*c*^(*s*_2_) = (3,2,6), *f*^*c*^(*s*_3_) = (3,2,3), *f*^*c*^(*s*_4_) = (2,3,4), …

*v*(*s*_1_) = (2,1,5), *v*(*s*_2_) = (2,1,5), *v*(*s*_3_) = (2,1,3), *v*(*s*_4_) = (1,2,4), …

From the above computation results, we can get that string *s*_1_ and string *s*_*2*_ belong to the same partition while string *s*_3_ and string *s*_4_ belong to different partitions.

## 4. Parallel processing in multi-core systems

In order to make full use of the capability of the multi-core system, we design and implement a parallel string join algorithm called Para-Join.

### 4.1 String join algorithm para-join

Algorithm 1 shows the pseudo code of Para-Join. Consider a string set *S*, it is firstly split into *S*' (*S*' = *S*_1_∪*S*_2_∪⋯∪*S*_*n*_) in parallel based by the frequency distribution function called *fqSplit*(·). Then a parallel cycling alternation method is used to deal with *S*'. Theorem 1 shows that our algorithm can eliminate the redundant computation and guarantee the completeness of the result. The major flow can be described as follows. Firstly the set S is split into n subsets and the corresponding threads are created. In each thread, function *para-RR*(·) is invoked to seek the similar string pairs of *S*_*j*_. For any subset *S*_*j*_, function *para-RS*(·) helps to search all the similar pairs between *S*_*i*_ and *S*_*j*_ (i<j).

**Algorithm 1: Para-Join**

**Input:** S     //A set of strings

      τ     //A given similarity threshold

**Output:** *ψ*  //*ψ* = {(*s*,*r* ∈ *S*)|*Sim*(*s*,*r*) ≤ *τ*}

**1**  **begin**

**2**    Main thread:

**3**      *S*'←fqsplit(S);  //split the set S into n subsets

**4**      *ψ*←Φ;

**5**      SubThread [] threads = new SubThread[*S*'.size()];  //create a thread array

**6**      for (j = 0; j<*S*'.size(); j++)

**7**        SubThread threads[j] = new SubThread(j);  //create n threads and run them

**8**        threads[j].start();

**9**      for (j = 0; j<*S*'.size(); j++)

**10**       threads[j].join();  //main thread waits for all of the processing threads to finish

**11**     for (j = 0; j<*S*'.size(); j++)

**12**       *ψ*←*ψ*∪threads[j].get();  // the union of the result produced by the n processing threads

**13**   Processing thread with parameter j:

**14**      *ψ*1←Φ; *ψ*2←Φ;

**15**      *ψ*1←*ψ*1∪para-RR(*S*'.get(j));

**16**       for (i = 0; i<j; i++) do

**17**         *ψ*2←*ψ*2∪para-RS(*S*'.get(i), *S*'.get(j));

**18**       return *ψ*1∪*ψ*2;

    **19  end**

#### Theorem 1

Para-Join can not only avoid repetitive computation but also ensure the completeness of the result.

#### Proof

Given a collection of strings *S*, we split it into n small subsets, *S*_1_,*S*_2_,⋯,*S*_*n*_. According to algorithm Para-Join, for any subset *S*_*j*_ (*j = 1*,*2*,*…*, *n*), we need to find the similar pairs between *S*_*i*_ and *S*_*j*_ (*i<j*). Because the value of *j* ranges from 1 to n, for any *S*_*i*_ and *S*_*j*_ (*i≠j*), the search processing will be executed only once. For each *S*_*i*_, the algorithm will search the similar pairs in *S*_*i*_ at first. So Para-Join will not miss any similar pairs, i.e., it can ensure the completeness of the result. Furthermore, there is no redundant similarity computation between any two strings in the algorithm. So we can see that Para-Join can also avoid repetitive computation.

## 4.2 Data partition and similarity computation

The function *fqSplit*(·) is designed for the data partition. Given a collection of strings S, there exist a lot of methods to split it into some small subsets. In this paper, we propose a parallel strategy which can achieve data partition in a shorter period of time. The pseudo-code is illustrated in algorithm 2. Firstly the frequency variance of each token in *Σ* is calculated. Then Σ is split into multiple subsets in parallel by Z-Collapsing algorithm. Each subset *Σ*_*i*_ is called a joint-token. For each string, its joint frequency vector is calculated, and for each joint-token, the range of the frequency distribution called range-frequency is also calculated. Finally, the function splits the string set *S* into subsets.

**Algorithm 2: fqSplit**

**Input:** *S*    //A given set of strings

**Output:** *S*'  //*S*' = {*S*_*i*_|∀*s*,*r* ∈ *S*_*i*_, *S*_*i*_ ⊂ *S*, *v*(*s*) = *v*(*r*)}

**1**  **begin**

**2**    for each token, calculate the variance of frequency in parallel;

**3**    Σ'←Paralleled divide *Σ* into several sets;

**4**    for each *s* (*s* ∈ *S*), paralleled calculates *f*^*c*^(*s*);

**5**    split the range of frequency distribution in parallel;

**6**    for each *s* (*s* ∈ *S*), paralleled compute *v(s)*;

**7**    *S*'←Partition *S* by using the multi-threading technique in parallel;

**8**    return *S*';

**9**  **end**

In section 2 the position filtering and the extension-based verification methods have been explained in detail. We design function *posFilter*(·) and function *verification*(·) to implement these two methods. If *posFilter(s*,*r*,*τ)* returns false, string *s* and string *r* are dissimilar. If *posFilter(s*,*r*,*τ)* returns true, pair *<s*,*r>* is added into the candidate set. Function *verification(s*,*r*,*τ)* returns the similarity of string *s* and string *r*. In this paper, we develop a pruning strategy by extending the position filtering to remove the dissimilar pairs. By utilizing this pruning strategy, we can get a smaller candidate set. Suppose *s* and *r* denote two different strings, *v(s)* and *v(r)* denote their interval-vector respectively. The following is the description of the process in two different cases.

1. *v(s) ≠ v(r)*. If function *posFilter(s*,*r*,*τ)* returns true and the inequality *dis(v(s)*, *v(r))≤2τ* is established, pair *<s*,*r>* can be added into the candidate set. If pair *<s*,*r>* is in the candidate set and inequality *verification(s*,*r*,*τ)≤τ is established*, we can get that *s* is similar to *r*.
2. *v(s) = v(r)*. If function *posFilter(s*,*r*,*τ)* returns true and the inequality *dis(v(s)*, *v(r))≤2τ* is established, pair *<s*,*r>* can be added into the candidate set. If pair *<s*,*r>* is in the candidate set, and the inequality *verification(s*,*r*,*τ)≤2τ is established*, we can also get that string *s* is similar to string *r*.

## 4.3 Join operation

Two functions named *para-RR* (·) and *para-RS*(·) are designed to do the join operation. The function *para-RR*(·) extends the partition-based algorithm and implements the self-join in a subset by using the multi-threading technique [2,4]. There are three main steps in *para-RR*(·):

Step 1: *S*_*i*_ is sorted by the string length in descending order.

Step 2: The inverted index *L*^*i*^_*l*_ is built. The variable *l* is the string length and the variable *i* is the index of the string segment.

Step 3: For any two strings, their similarity is calculated by adopting the above method. For example, given two strings *s* and *r*, function *para-RR*(·) first computes their joint-frequency vectors *f*^*c*^(*s*) and *f*^*c*^(*r*). If the *L*_*1*_ distance of their joint-frequency vector is larger than 2*τ*, we can get that these two strings are dissimilar. Otherwise, it will check the string pair <*s*,*r*> by invoking function *verification*(·).

The function *para-RS*(·) primarily focuses on how to find the similar pairs between two different collections.

Given two different sets *S*_*i*_ and *S*_*j*_; *v(S*_*i*_*)* and *v(S*_*j*_*)* denote the IDs of *S*_*i*_ and *S*_*j*_ respectively. If *dis(v(S*_*i*_*)*,*v(S*_*j*_*))* is larger than *2τ*, it shows that they cannot be matched successfully. If *dis(v(S*_*i*_*)*, *v(S*_*j*_*))* is not larger than *2τ*, the function *para-RS*(·) can find the similar pairs by employing the above pruning strategy. For example, given a string *r* in *S*_*i*_, for any string *t* in *S*_*j*_ (*l*_*min*_ ≤ *length(t)* ≤ *l*_*max*_)), the function first checks whether *r* and *t* are similar by function *posFilter*(·), and then it calculates the *L*_*1*_ distance of joint-frequency vector of *r* and *t*.

## 5. Parallel processing in distributed systems

A big problem for parallel processing with multi-threading technique is the incapability of the system such as the limited memory and the number of cores. One solution is to add the memory and the other solution is to run the framework in a distributed cluster environment.

### 5.1 String join algorithm pada-join

Hadoop as a big data processing technology has been around for 10 years and has proven to be the solution of choice for processing large data sets. MapReduce is a great solution for one-pass computations, but not very efficient for use cases that require multi-pass computations and algorithms. Each step in the data processing workflow has one map phase and one reduce phase and the developers will need to convert any use case into MapReduce pattern to leverage this solution.

Spark allows programmers to develop complex, multi-step data pipelines using directed acyclic graph (DAG) pattern. It also supports in-memory data sharing across DAGs, so that different jobs can work with the same data. Spark runs on top of existing HDFS infrastructure to provide enhanced and additional functionality.

We propose a parallel string join algorithm called Pada-Join based on Spark. Algorithm 3 shows the pseudocode of Pada-Join, where the bold functions or methods are provided by Spark.

**Algorithm 3: Pada-Join**

**Input:** *S*    // A set of strings

       *τ*    // A given similarity threshold

**Output:** *ψ*//*ψ* = {(*s*,*r* ∈ *S*)|*Sim*(*s*,*r*) ≤ *τ*}

**1** **begin**

**2   Map**(<rid,string>); //the filter stage

**3**   compute joint-frequency vector f(r) for string r;

**4**   emit(<f(r), rid>);

**5   Groupbykey**(<f(r), rid>);

**6**   emit(<f(r),list(rid)>);

**7**   <f(s),list(sid)>←broadcast(<f(r),list(rid)>);

**8   MapPartitions**(<f(r),list(rid)>,<f(s),list(sid)>);

**9**   for <f(ri),list(rid)> in <f(r),list(rid)>

**10**     for <f(si),list(sid)>) in <f(s),list(sid)>

**11**       if dis(f(ri), f(si)) ≤ 2τ then emit(<list(rid),list(sid)>);

**12   Flatmap**(<list(rid),list(sid)>);

13   for rid in list(rid) emit(<rid,list(sid)>);

**14   Join**(<rid,list(sid)>,<rid,r>);

**15**   emit(<r,list(sid)>);

**16   Flatmap**(<r,list(sid)>);

17   for sid in list(sid) emit(<sid,r>);

**18   Join**(<sid,r>,<sid,s>);

**19**   emit(<s,r>);

**20   Filter**(<s,r>);

**21**   if  Sim(s,r)≤τ then emit(<<s,r>,Sim(s,r)>);

      **22    end**

The joint frequency vector *f(r)* for each string of the given dataset is generated in the filter stage. In order to get the joint frequency vector, we need to obtain the token set according to the token counting algorithm. Algorithm 4 shows the pseudocode of how to compute the token set, where the bold functions or methods are provided by Spark.

If two strings are similar, the distance of their joint-frequency vectors must be less than 2*τ*. The candidate pairs are produced by utilizing the cartesian product of all the distinct pairs of the distribution nodes. However, this operation takes a huge amount of memory to store all the pairs that are distributed across multiple machines. To minimize the size of pairs, the vectors are taken as keys and the string IDs are taken as values so that the pairs sharing the same joint-frequency vector are assigned to the same group (seen in lines 5–6 of algorithm 3). The lines 7–13 in algorithm 3 illustrate the stage of candidate generation. To reduce data communication and data shuffling among the nodes, we store the joint frequency vector groups <*f(r)*,*list(rid)*> in memory by generating a broadcast variable <f(s),list(sid)>. Then the candidate groups to meet the filtering condition are matched.

In the verification phase, *rid* and *sid* need to be converted into string *r* and *s*, and then to be verified. Line 14 does the job by joining dataset *S* with *<rid*,*list(sid)>*. The variable *sid* coming from the broadcast *<f(s)*,*list(sid)>* is generated from <*f(r)*,*list(rid)*>. Then the candidate pair <*s*,*r*> can be obtained by joining the dataset *S* with <*sid*,*r*> (seen in lines 18–19), and they are matched by calculating their similarity. The output is the final result.

**Algorithm 4: token count**

**Input:** *S*    //A given set of strings

**Output:** *S*'  //*S*' = {*S*_*i*_|∀*s*,*r* ∈ *S*_*i*_, *S*_*i*_ ⊂ *S*, *v*(*s*) = *v*(*r*)}

**1** **begin**

**2   Flatmap**(<rid,string>);

**3**   for each token in the string r emit(<token,1>);

**4   Reduce**(<token,list(1)>);

**5**   emit(<token, tokenfrequency>);

**6**   *S*'←Z-folder(<tokenid, tokenfrequency>);

**7   return** *S*';

**8**      **end**

Because Pada-Join and Para-Join share the same algorithmic logic, Pada-Join can also avoid repetitive computation and ensure the completeness of the result.

### 5.2 Join operation in spark

The following shows the computation flow of join operation in Spark.

**Fig 2:**
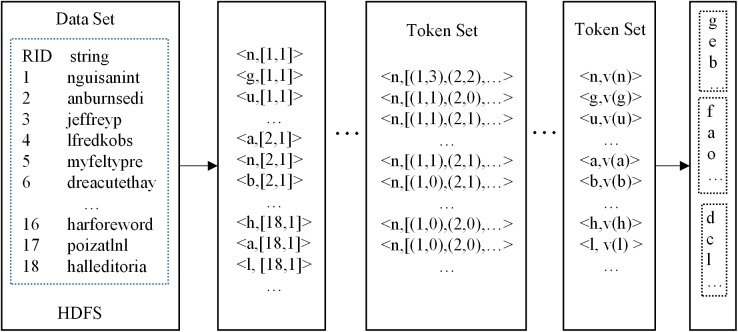
An instance to get the token set.

1) To get the token set dynamically and by partitioning. In Para-Join, the token set is obtained in advance. In Pada-Join, the token set is obtained dynamically. Fig 2 is an instance to get the token set.

After getting the token set, we need to split it into subsets. The partitioning rule is the same as that in Para-Join algorithm, i.e., calculating the frequency distribution for each token and the frequency variance, and then getting the token set according to the Z-folder algorithm.

**Fig 3:**
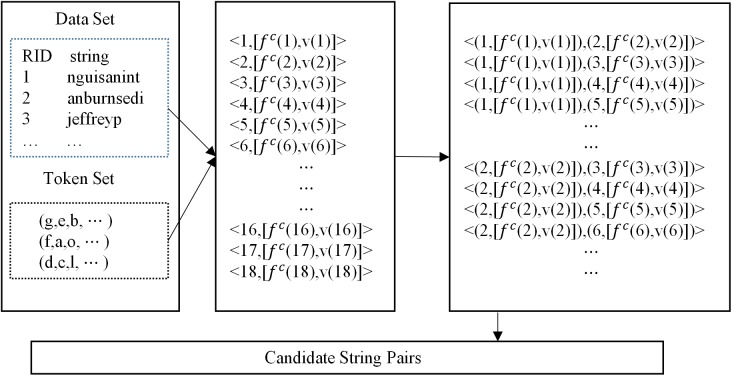
An instance to get the candidate string pairs

2) To get the candidate string pairs by filtering. Then delete the string pairs that are impossible similar. The way is the same as that in Para-Join algorithm. Fig 3 shows an instance to get the candidate string pairs.

3) To get the result by verification. The verification process is the same as that in Para-Join algorithm.

## 6. Experimental evaluation

### 6.1 Experimental environment

In this section, we evaluate the parallel string join algorithms based on the real datasets. Four datasets are used in the experiment. All the datasets can be downloaded from http://doi.org/10.5281/zenodo.293041. Dataset Ⅲ and Ⅳ can be downloaded from http://dbgroup.cs.tsinghua.edu.cn/dd/codes/pivotal.tar.gz too.

The first two datasets are relatively small and used to test the single-machine algorithms. The detailed information of these datasets is shown in Table 2.

**Table 2:** Datasets information.

Datasets	Cardinality	Avg Len	Max Len	Min Len
dataset I: Author+Title	863,073	105.82	886	21
dataset Ⅱ: AOL Query Log	464,189	44.75	522	30
dataset Ⅲ: URL	1,000,000	28.03	193	20
dataset Ⅳ: DNA	2,476,276	108.0	108	108

Algorithms Pass-Join, Part-Join, and Para-Join are implemented in Java, and Algorithm Pada-Join is implemented in Scala. These algorithms run on three different systems: a multi-core system, a cluster system with 4 nodes, and a single machine with the same configuration as the node in the cluster. The operating system used is Ubuntu 12.04 LTS and the version of JDK is 1.7.0_71. The detailed information of the systems is shown in Table 3 where system Ⅱ is consisted of 4 nodes and they are virtual machines. The virtual machines are created on a physical hardware with CPU i7-4770 3.40 GHz *8, RAM 16GB, and hypervisor VMware.

**Table 3:** System information.

System	node number	CPU	RAM
system I: multi-core system	1	i7-4770 3.40 GHz *8	16GB
system Ⅱ: cluster system	4	i7-4770 3.40 GHz*2	3GB
system Ⅲ: single machine	1	i7-4770 3.40 GHz*2	3GB

We evaluate our framework in two aspects, efficiency and scalability.

In efficiency aspect we evaluate the running time of parallel processing with multi-threads and multi-tasks against the existing algorithms.

In scalability aspect we evaluate the influence of the number of threads or tasks attending the computation.

### 6.2 Efficiency analysis

**Fig 4:**
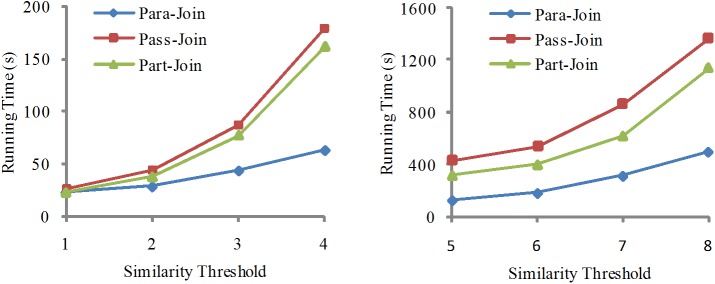
Running time comparison among different algorithms for dataset I in system I.

**Fig 5:**
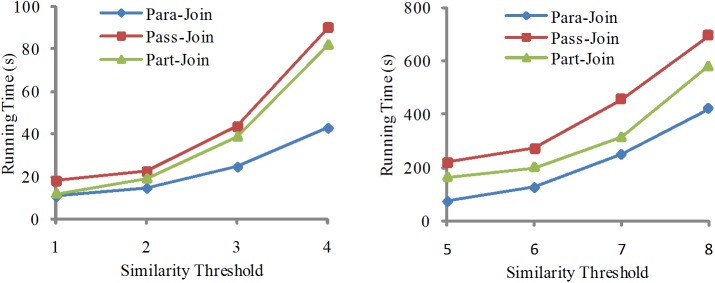
Running time comparison among different algorithms for dataset II in system I.

In this Section, we compare our algorithms with two existing algorithms Pass-Join and Part-Join. For Para-Join, the number of threads is set to 8. The similarity threshold *τ* ranges from 1 to 8. The experimental results are shown in Figs 4 and 5. Because similarity thresholds have high influence on the running time, the results are shown in different figures with varying thresholds.

When the similarity threshold *τ* is small, there is no big difference for the running time among algorithms. For example, when *τ* is 1, the running times of the three algorithms on dataset I are 22s, 25s, and 23s respectively. When the value of τ increases, algorithm Para-Join can show more advantages. For example, when τ is 8, the running time on dataset I of Para-Join is 49s while the others are 136s and 114s respectively. It maintains the same advantage on dataset II. The main reason is that our algorithm can concurrently find the similar pairs in the dataset by using the multi-threading technique.

**Fig 6:**
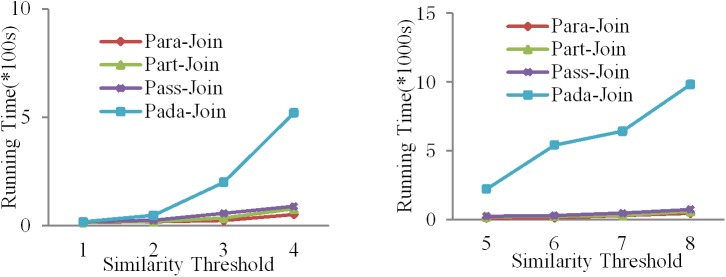
Running time comparison among different algorithms for dataset II in system Ⅱ and system Ⅲ.

When we test dataset Ⅱ in system Ⅱ and system Ⅲ, the running time is bigger than in other algorithms. Fig 6. shows the results. We realize that Pada-Join is not suitable for small datasets.

When we test dataset Ⅲ and dataset Ⅳ in system Ⅲ, the memory overflow error occurs. However, Pada-Join completes the work successfully. We also realize that Para-Join, Pass-Join, and Part-Join are not suitable for big datasets.

For Para-join algorithm, the implementation is to load the input into memory at first and then to process it. For Pada-join, the Spark framework will divide the input into several blocks and store them in the HDFS (Hadoop distributed file system). The size of a block is limited and it can be loaded into memory at the same time. After Spark finishes the processing of one block, it will load another block. The basic differences between Para-join and Pada-Join are their implementation ways and the platforms they run on. So Para-Join algorithm is unable to handle the larger dataset.

### 6.3 Scalability analysis

We have designed two cases to evaluate the scalability of the algorithms Para-Join and Pada-Join.

#### Case 1

**Fig 7:**
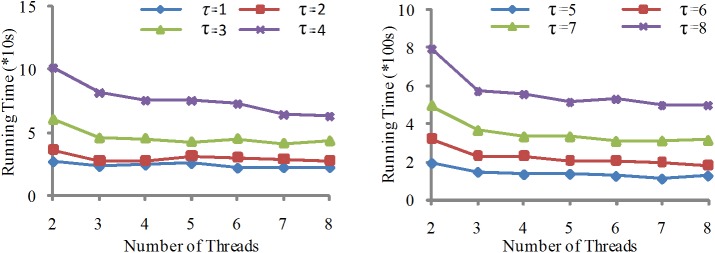
Running time comparison of Para-Join for dataset I in system I.

**Fig 8:**
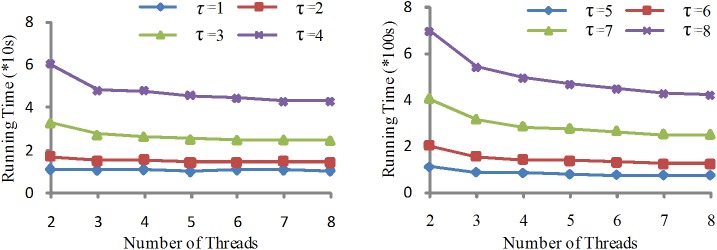
Running time comparison of Para-Join for dataset II in system I.

Under the same dataset, we compare the running times by changing the number of threads from 2 to 8 and changing the similarity threshold *τ* from 1 to 8. The experimental results are shown in Figs 7 and 8.

From the figures, we can observe that the running time increases as the value of *τ* increases. The reason is that for the same dataset, when *τ* increases, candidate pairs in the dataset are also increased, result in more operations in the verification process. However, when the value of *τ* is big enough, e.g., *τ* is 8, the running time remains unchanged or even becomes larger. The reason is that a large number of threads increase the communication overhead.

#### Case 2

**Fig 9:**
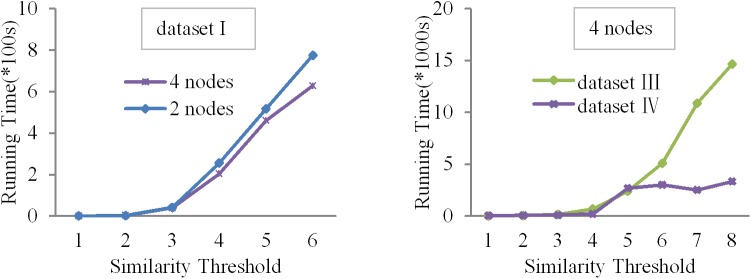
Running time comparison of Pada-Join among different similarity threshold in system II.

Under the same system configuration, we compare the running times for dataset Ⅲ and dataset Ⅳ by changing the similarity threshold *τ* from 1 to 8. These two datasets are too large to the extent that the other algorithms cannot handle. As *τ* becomes larger, the algorithm becomes more complicated. Because the number of the candidate pairs increases with the size of datasets, the running time also increases. The experimental results are shown in Fig 9. The algorithms can perform excellently on larger data sets.

## 7. Related work

There are many previous studies on the development of efficient solutions to the string similarity join problem [1–22].

### String similarity functions

The string similarity functions are the key for all the string similarity join algorithms. String similarity functions are used to quantify the similarity of two strings. The existing string similarity functions can be roughly divided into two groups, character-based similarity, and set-based similarity. The character-based similarity considers characters in strings to quantify the similarity, such as Edit distance, Hamming distance, and character n-gram similarity [6,13,14]. The set-based similarity quantifies the similarity based on the token sets. These functions include Jaccard similarity, Cosine similarity, and Dice similarity [13,14]. Besides the above similarity functions, there are also some new functions, such as Jaro-Winkler measure and Hidden Markov Mode-based measure.

### String similarity join methods

The existing methods for string similarity join can be broadly separated into two categories, based on the filtering-verification framework and the tire tree. Most of the existing methods adopt the first one. These methods include All-Pairs-Ed, ED-Join, AdaptJoin, Part-Enum, Pass-Join, and Part-Join [2,3,8,15–17]. All-Pairs-Ed is a q-gram-based method, ED-Join improves All-Pairs-Ed using location-based and content-based mismatch filter by decreasing the number of grams, and AdaptJoin algorithm improves the prefix filtering fundamentally for all similarity metrics. Trie-Join and Bed-Tree use a trie tree to do similarity join [12,11]. With the improvement of these methods, many filtering techniques are proposed such as count filtering, length filtering, position filtering, prefix filtering, and content filtering [1,2,4,10,17,18]. Additionally, some parallel methods have been proposed for string similarity join, such as bit-parallel, MassJoin, V-SMART-Join, et al [7,18,19].

### String similarity search

It is similar to string similarity join. Firstly the index of the string collection is built. When a query request is submitted, a large number of dissimilar strings are filtered according to the given query string, and then the candidate strings are matched with the given string according to the similarity function [4,11,20].

### Parallel processing techniques

There are a lot of works on implementing string join using Map-Reduce framework. Vernica et al. proposed a similarity join method using MapReduce which utilized the prefix filtering to support set-based similarity functions [16]. They selected a subset of tokens as signatures and proved that two strings are similar only if their signatures share common tokens. Afrati et al. proposed multiple algorithms to perform similarity joins in a single MapReduce stage [21]. They analyzed the map, reduce, and communication cost. However, for long strings, it is rather expensive to transfer the strings using a single MapReduce stage. Kim et al. addressed the top-k similarity join problem using MapReduce [22]. Deng et al proposed Mass-Join which extended the existing partition-based signature scheme to support set-based similarity functions [11]. In this paper, we take the multi-threading technology and the multi-tasking technology into consideration and compare them in the string join field.

## 8. Conclusions

In this paper, a parallel processing framework for string similarity join is proposed for high efficiency. Algorithm Para-Join based on the framework adopts the multi-threading technique and runs on the multi-core system. Algorithm Pada-Join, also based on the framework, adopts the distributed computing technique and runs on the distributed systems. Some conclusions are given by the experimental results and analysis. For relatively small datasets Para-Join can provides very good scalability and outperforms state-of-the-art algorithms because it completes the computation of string similarity join in one node and avoids the overhead of network communication. However, the availability of single-machine algorithms is limited by the memory. For relatively big data set, Pada-Join shows its advantages because of the good scalability of the distributed systems. In the future, we will adopt larger datasets to test Pada-Join algorithm and improve its performance.

## References

[1] Jiang Y, Li G, Feng J, Li W. String similarity joins an experimental evaluation. Int. Conf. on Very Large Data Bases (VLDB). 2014; 7(8): 625–636.

[2] Li G, Deng D, Wang J, Feng J. Pass-Join: a partition-based method for similarity joins. Int. Conf. on Very Large Data Bases (VLDB). 2012; 5(3): 253–264.

[3] Chen Y, Luo J, Li J. Part-Join: partition based string similarity join. Application Research of Computers (in Chinese), 2014; 10: 3002–3006.

[4] Jiang Y, Deng D, Wang J, Li G, Feng J. Efficient parallel partition-based algorithms for similarity search and join with edit distance constraints. Joint EDBT/ICDT 2013 Workshops. 2013; March 22: 341–348.

[5] Jestes J, Li F, Yan Z, Yi K. Probabilistic string similarity joins. ACM Int. Conf. on Management of data (SIGMOD). 2010; June 6–11: 327–338.

[6] Wang J, Li G, Feng J. Extending string similarity join to tolerant fuzzy token matching. ACM Transactions on Database Systems (TODS). 2014; 39(1): 7.

[7] Xu K, Cui W, Hu Y, Guo L. Bit-parallel multiple approximate string matching based on GPU. Procedia Computer Science. 2013; 17: 523–529.

[8] Wang J, Li G, Feng J. Can we beat the prefix filtering? An adaptive framework for similarity join and search. ACM Int. Conf. on Management of Data (SIGMOD). 2012; May 20–24: 85–96.

[9] Alba A, Rodriguez-Kessler M, Arce-Santana ER, Mendez MO. Approximate string matching using phase correlation. Annual Int. Conf. of the IEEE Engineering in Medicine and Biology Society (EMBC). 2012; August 28-September 1: 6309–6312. 10.1109/EMBC.2012.6347436 23367371

[10] Xiao C, Wang W, Lin X, Yu J. Efficient similarity joins for near-duplicate detection. ACM Trans. Database Syst. 8 2011; 8: 1–41.

[11] Zhang Z, Hadjieleftheriou M, Ooi B, Srivastava D. Bed-Tree: an all-purpose index structure for string similarity search based on edit distance. ACM Int. Conf. on Management of Data (SIGMOD). 2010; June 6–11: 915–926.

[12] Wang J, Feng J, Li G. Trie-Join: Efficient trie-based string similarity joins with edit-distance constraints. Int. Conf. on Very Large Data Bases (VLDB). 2010; 3(1): 1219–1230.

[13] Lu J, Lin C, Wang W, Li C, Wang H. String similarity measures and joins with synonyms. ACM Int. Conf. on Management of Data (SIGMOD). 2013; June 22–27: 373–384.

[14] Wang J, Li G, Fe J. Fast-Join: An efficient method for fuzzy token matching based string similarity join. IEEE 27th Int’l Conf. on Data Engineering (ICDE). 2011; April 11–16: 458–469.

[15] Bayardo RJ, Ma Y, Srikant R. Scaling up all pairs similarity search. 16th Int. World Wide Web Conference (WWW). 2007; May 8–12: 131–140.

[16] Vernica R, Carey M, Li C. Efficient parallel set-similarity joins using mapreduce. ACM Int. Conf. on Management of data (SIGMOD). 2010; June 6–11: 495–506.

[17] Wang W, Xiao C, Lin X, Zhang C. Efficient approximate entity extraction with edit distance constraints. ACM Int’l Conf. on Management of data (SIGMOD). 2009; June 29-July 2: 759–770.

[18] Metwally A, Faloutsos C. V-smart-join: A scalable mapreduce framework for all-pair similarity joins of multisets and vectors. Int. Conf. on Very Large Data Bases (VLDB). 2012; 5(8): 704–715.

[19] Deng D, Li G, Hao S, Wang J, Feng J. MassJoin: a mapreduce-based method for scalable string similarity joins. IEEE 30th Int. Conf. on Data Engineering (ICDE). 2014; March 31-April 4: 340–351.

[20] Yuan X, Long J, Zhang Z, Gui W. Near-duplicate document detection with improved similarity measurement. Journal of Central South University. 2012; 9(8): 2231–2237.

[21] Afrati FN, Sarma AD, Menestrina D, Parameswaran AG, Ullman JD. Fuzzy joins using mapreduce. IEEE 28th Int. Conf. on Data Engineering (ICDE). 2012; April 1–5: 498–509.

[22] Kim Y, Shim K. Parallel top-k similarity join algorithms using mapreduce. IEEE 28th Int. Conf. on Data Engineering (ICDE). 2012; April 1–5: 510–521.

